# Intranasal esketamine as therapeutic option: a case report of an adolescent with treatment resistant depression

**DOI:** 10.3389/fpsyt.2023.1118737

**Published:** 2023-06-02

**Authors:** Katrin Skala, Kamer Doganay, Harald Eder, Dunja Mairhofer, Katrin Neubacher, Paul L. Plener

**Affiliations:** ^1^Department of Child and Adolescent Psychiatry, Medical University Vienna, Vienna, Austria; ^2^Department of Child and Adolescent Psychiatry and Psychotherapy, University of Ulm, Ulm, Germany

**Keywords:** ketamine, adolescents, intranasal, treatment resistant depression, off-label

## Abstract

Depression is among the most common mental health disorders worldwide and treatment resistant depression (TRD) represents a major challenge for both patients and clinicians. In recent years ketamine has received attention as an antidepressant agent, demonstrating promising results in TRD in adults. To date, few attempts have been made in treating adolescent TRD with ketamine and none have used intranasal application. This paper discusses a case of a 17-year-old female adolescent suffering from TRD who underwent treatment with intranasal esketamine application (Spravato 28 mg). As symptoms showed clinically insignificant improvement despite modest gains in objective assessments (GAF, CGI, MADRS), treatment was prematurely discontinued. However, the treatment was tolerable and side effects were scarce and mild. Although this case report does not demonstrate clinical effectiveness, ketamine may nonetheless be a promising substance in treating TRD in other adolescents. Questions regarding the safety of ketamine use in the rapidly developing brains of adolescents still remain unanswered. To further explore the potential benefits of this treatment method a short term RCTs for adolescents with TRD is recommended.

## Introduction

Major Depressive Disorder is among the most common mental health disorders worldwide, both in adults, as well as in adolescents. A recent meta analysis reported a global point prevalence of depressive symptoms from 2001 to 2020 of 34% in youth with a point prevalence for major depressive disorder of 8% (1). Throughout the COVID-19 pandemic, these rates are reported to have increased, where, for example, a meta-analysis indicated clinically elevated depressive symptoms in 25.2% of youth (2) highlighting the need for effective evidence-based treatment options.

However, not every youth responds favorably to this treatment approach and nearly 40% of adolescents present with clinically significant depressive symptoms following initial treatment (3), thereby exhibiting a degree of treatment resistance. Definitions of treatment-resistant depression (TRD) vary: For adults, the most common definition is failure of two or more antidepressant medications given at adequate doses for 6–8 weeks during a major depressive episode. Dwyer et al. (3) have proposed that this category should extend to youth with clinically significant symptoms of depression after a single trial “of an evidence-based psychotherapy and an antidepressant with Grade A evidence for treating depression in pediatric population (fluoxetine, escitalopram, or sertraline).”

While there is little research examining effective treatment methods for adolescents, a staging model has been proposed by Dwyer et al. (3) in which an SSRI is added to psychotherapy. The pharmacological treatment is raised to the maximally tolerated dose before it is replaced by an alternate SSRI and—in the case that no significant effect is achieved—it is combined with alternate antidepressants or augmentation strategies proven to be effective in adult samples (e.g., antipsychotics, lithium, bupropion, mirtazapine or stimulants). Following an escalation approach, this scheme can be further complimented by interventional treatments (such as repetitive transcranial magnetic stimulation), ketamine or electroconvulsive therapy in the highest stage (3, 4).

In recent years ketamine has received attention as an antidepressant agent, showing promising results for TRD in adults (9) as well as having anti-suicidal properties (10). Ketamine is an N-methyl-D-aspartate receptor antagonist with the enantiomers arketamine and esketamine (7). While esketamine has been found to be effective for use in adults with TRD and was thus approved for TRD in adults in several countries, use of this psychopharmacological agent in adolescents has received less attention. A recent review on the therapeutic use of ketamine in children and adolescents identified four studies describing ketamine use by intravenous and subcutaneous application in TRD in minors while naming three additional studies not fulfilling eligibility criteria (11). Results indicate that ketamine improved depressive symptoms, decreased acute suicidality, and reduced mood lability, though a number of subjects remained resistant to its treatment (11). A search of PubMed for “ketamine or esketamine and TRD” yielded five additional publications presenting results from intravenous or subcutaneous use of ketamine in adolescents with TRD and describing a significant reduction in depressive symptoms (7) (see Table 1). A review of existing literature on alternate application methods of ketamine yielded a case series of intranasally administered ketamine in ten males and two females between the ages of six and 19 for the treatment of bipolar disorder. Minimal side effects and clinical improvement were reported (12). As a follow-up to this research, the same group presented data of 45 patients diagnosed with bipolar disorder (both youth and adults between 6 and 37 years with a mean age of 15) who received intranasal ketamine treatment with varying treatment lengths. Results indicate significant reductions of symptoms and (mild) persistent adverse events in 13 cases (13).

**Table 1:** Studies on ketamine application minors with TRD, based on own PubMed search (FU, follow-up).

**References**	**Age**	**Study type**	**Number of participants**	**Intervention**	**Finding**
Cullen et al. (5)	12–18	Case-series	13	Six ketamine (0.5 mg/kg) infusions over 2 weeks	Average decrease in CDRS-R: 42.5% (*p* = 0.0004). Five (38%) adolescents: clinical response., three responders: sustained remission at 6-week FU
Dwyer et al. (6)	16	Case report	1	Seven infusions over an 8-week hospitalization (days 1, 3, 7, 14, 21, 28, 50).	Rapid reduction in depressive symptoms on first day (61% MADRS reduction; 32% CDRS reduction), treatment gains intensified and persisted
Dwyer et al. (3)	13–17	Randomized, doubleblind, single-dose crossover clinical trial,	17	Single intravenous infusion of either ketamine or midazolam, change to alternate compound 2 weeks later.	Single ketamine:infusion significantly reduced depressive symptoms 24 h after infusion compared with midazolam; (treatment gains remain 14 days Revised). Greater response to ketamine during the first 3 days vs. midazolam (76 and 35%, respectively).
Faria-Guimarãest et al. (7)	Mean age: 15.5 (±1.35)	Case series	10	Single application: 8 patients received subcutaneous esketamine, 2 patients intravenous esketamine	Significant reduction in depressive symptoms (mean total MADRS score) from baseline to 24-h postadministration (mean difference = 12.3; *t* = 4.22; *p* = 0.002; *d* = 1.33).
Zarrinnegar et al. (8)	15	Case report	1	Six ketamine infusions dosed at 0.5 mg/kg per infusion for the course of 3 weeks	Gradual decreases in depressive symptoms (Montgomery–Asberg Depression Rating Scale, Children's Depression Rating Scale)

A recent online survey of 283 parents explored attitudes regarding the use of ketamine in adolescents and results showed that the use of ketamine for suicidality, bipolar disorder and major depressive disorder in minors exhibited high acceptability among respondents (14). Furthermore, parents reported a preference for less invasive administration modes, with nasal spray as the most preferred application method (14).

Ketamine seems to be a promising substance for the treatment of various affective disorder, yet literature on its use in adolescents is scarce and to our knowledge, no study has sofar investigated intranasally administered ketamine in adolescents.with TDR.

In this paper we describe a therapeutic trial with intranasal ketamine in an adolescent with TRD.

## Case study of intranasal esketamine treatment with an adolescent female

M, a 17-year-old Caucasian female student was admitted to our acute psychiatric unit due to chronic suicidal ideation. Suicidal thoughts first emerged at the age of 15 and these had worsened in the weeks prior to admission. She reported symptoms of anxiety beginning 6 years of age and these were present with fluctuations since then. Fears initially revolved around climate catastrophes and war. She currently describes social anxiety and the fear of the death of her parents. At the outset of symptoms she struggled with diurnal incontinence and difficulties initiating and maintaining sleep, which led to seeking mental health treatment. She then attended psychotherapy for 2 years during primary school. Several months prior to hospitalization she first began self-harming. Non-suicidal self-injurious behavior included bruising and scratching her legs and burning her forearms with matches. Prior to intake at the Child and Adolescent Psychiatry (CAP) she had started collecting prescription medication with the intention of taking her own life.

Prior to the worsening of symptoms, the patient had been a good student, with the exception of some difficulties with math. Her parents described gradually emerging difficulties in the school setting. The patient spent more and more time writing excessive lists and planning elaborate systems to become more organized, but was often unable to sustain these goals and reverted to creating new organizational systems.

During the COVID- lockdown in spring 2020, her mental state deteriorated. She spent increasing amounts of time in bed and struggled to motivate herself to do her chores. Her condition continued to worsen in the course of the next school year, specifically during the lockdown in autumn of 2020. Her performance at school significantly deteriorated during the months preceding intake. It proved to be increasingly difficult for her to concentrate and to complete the required tasks; ongoing symptoms of depression and anxiety made it more and more difficult to attend school. She resumed psychotherapy and was able to complete the first term in school, and then failed to participate during the second term. At this point she withdrew, spending her days on the sofa and sleeping. She also gradually withdrew from her friends and ceased all contact a few weeks prior to admission.

In the course of the intake, no history of other psychological disorders, head injury or implantation, seizures, or substance abuse were identified. Additional, her thyroid function test and lab result, EEG and cerebral MRI results were found to be normal.

A family history of depressive disorders in both maternal and paternal relatives were reported. Both grandmothers had suffered from major depressive disorder, one uncle had made several suicide attempts, a second cousin had died by suicide, and her mother had struggled with postpartum depression after the patient's birth. At the time of admission, M was living with both her parents as well as her 10 year old brother.

During her inpatient treatment, the patient was also diagnosed with Asperger syndrome, generalized anxiety disorder and major depressive disorder. She exhibited traits of borderline personality disorder but did not meet the full diagnostic criteria.

Prior medication trials included an adequate dose of Sertraline (200 mg/day), Fluoxetine (60 mg/day), Alprazolam (in varying dosages), Venlafaxin (300 mg/day) and Quetiapin (600 mg/day), with Chlorprothixen (100 mg/day), Lorazepam (up to 10 mg/day)and Pregabalin (400 mg/day) as augmentation treatment, each for an adequate duration. After exhibiting refractory responses to various medications, she had attempted to overdose, presumably while experiencing mood congruent auditory hallucinations. Her persistent depressed mood and increased suicidal ideation despite therapy with three different antidepressants in sufficient dosage and over a sufficient period of time resulted in the attempt to administer esketamine.

A nasally administered formulation of esketamine has been approved for treatment of TRD in the adult population. Given the convenience of administration and the possibility of self-administration of the medication (allbeit under close medical supervision), we administered esketamine intranasally as part of an off-label use using the same nasal spray formulation that is approved for adults.

While psychotherapy continued, she received esketamine 28 mg nasal spray (Spravato) according to protocol (28 mg 2 × /week with 2 days interval for 4 weeks, then 28 mg once a week for 3 weeks). Medication was applied by a doctor for the first time, then by nurses. The patient remained under close supervision (ECG, blood pressure, saturation) for 1 h after each application.

Side effects included dizziness, which was present after most administrations, occasional nausea and fatigue. All side effects were of short duration (usually 30–60 min). Symptoms of derealization were experienced exclusively after the second administration and lasted for several hours. After one single dose a slight elevation of blood pressure, which also rapidly normalized, was observed.

Clinician ratings were assessed by a clinical psychologist not involved in the treatment of the patient.

To evaluate depressive symptomatology, we used Beck Depression Inventory, (BDI-II) (15) and the Patient Health Questionnaire (PHQ) (16) for self-report and the Montgomery-Asberg Depression Rating Scale (MADRS) (17) for clinician assessment. The severity of mental dysfunction and the general level of functioning were assessed with the Clinical Global Impression Scale (CGI) (18) and the Global Assessment of Functioning Scale (GAF) (19). CGI is a seven-point scale, that requires the clinician to rate the severity of the patient's illness at the time of assessment, relative to the clinician's past experience with patients who have the same diagnosis while GAF describes how much a person's symptoms affect their day-to-day life on a scale of 0–100.

**Figure 1:**
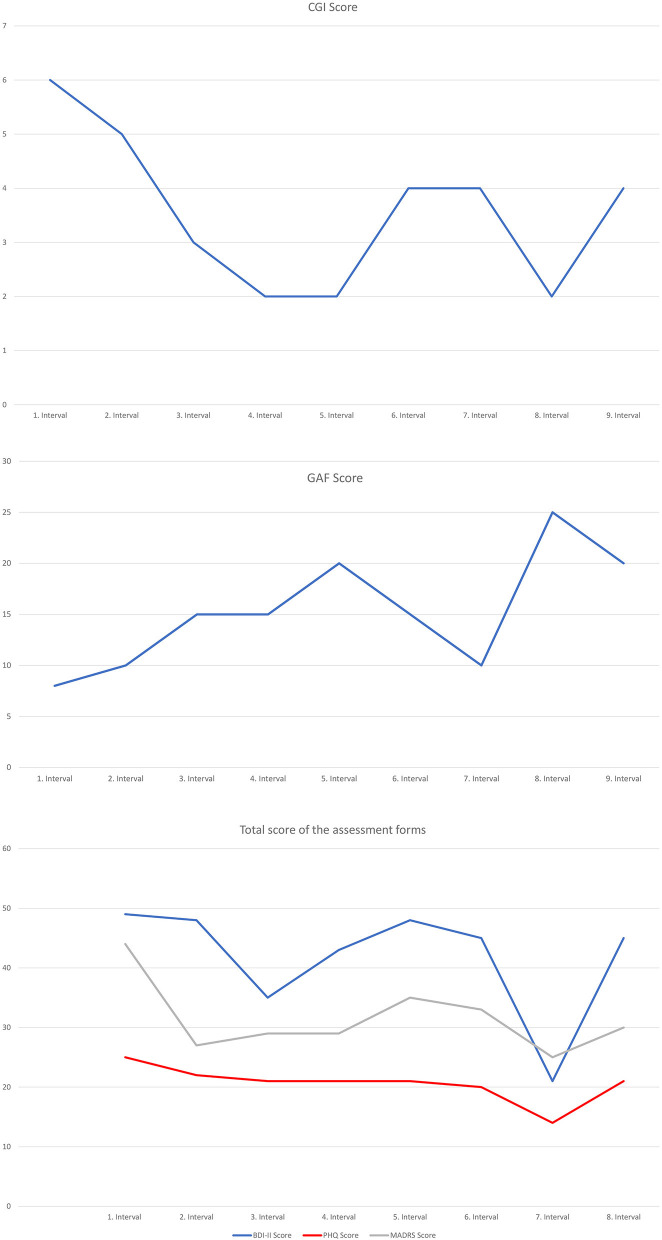
Self- and clinician administered rating forms. CGI, Clinical Global Impression Scale; GAF, Global Assessment of Functioning; BDI-II, Beck Depression Inventory; PHQ-9, Patient Health Questionnaire; MADRS, Montgomery-Asberg Depression Rating Scale.

On global scales, an improvement was noted: CGI decreased from 6 (very severe) to 4 (moderately severe)while GAF increased from a raw score of 8 to a score of 20. The results of the ratings of depressive symptomatology differed with regard to self vs. clinician rating. While the self-report measures (BDI-II PHQ-9) remained relatively unchanged (BDI-II: from raw score 49–45; PHQ-9: from raw score 25–21), MADRS showed a significant decrease (from a raw score of 44–30) in depressive symptomatology (see Figure 1).

No sustained improvement in mood or drive were however identified in the clinical observation. Likewise, no clinically significant changes in anxiety symptoms or in the general level of functioning were noted in subjective ratings and observation.

While tolerability was very good, and psychological evaluation (BDI, MADRS, PHQ) indicated some improvement, subjective ratings and clinical observation showed no clinically relevant persistent change of symptoms. Consequently, treatment was not continued.

## Discussion

In light of the high burden of insufficient treatment methods for TRD and the potential adverse effects, it is crucial to complement research on the efficacy of ketamine with research on potential markers for outcome prediction. Using an fMRI approach in 11 adolescents, Roy et al. (20) reported a greater increase in nucleus accumbens entropy in responders (*n* = 5). Within the same sample, better performance in the Word Face Stroop fMRI task (evaluating affective word superimposed on emotionally congruent or incongruent faces) correlated with decreased depressive symptoms, which could point to the direction of an attenuated negativity bias in adolescents responding to treatment (21).

Although the published reports seem promising in offering a potentially efficacious option for adolescents with TRD, questions about the safety of ketamine use in the rapidly developing brains of adolescents still remain unanswered. It has been pointed out that long-term consequences of repeated ketamine use in adolescents are not sufficiently studied and—given that depressive states could also represent a premorbid psychotic symptom—the risk of administering ketamine in vulnerable patient populations is as yet unknown (22). Developing parvalbumin interneurons depend on excitatory input for maturation, meaning that blocking NMDA currents might influence developmental pathways (22). Other clinical data link early life ketamine exposure to neural deficits, such as decreased GMV in the right insula, left inferior parietal lobule, left dorsolateral prefrontal cortex/superior frontal gyrus, left medial orbitofrontal cortex, or frontal white matter abnormalities (23, 24).

These findings call for further well-controlled RCTs in the field of adolescent TRD. So far, there is only one available RCT comparing single administration of ketamine and midazolam, showing a greater reduction of depressive symptoms after ketamine application that lasted up to 14 days (25). Building on these findings, a next step would be to consider short term RCTs for adolescents with TRD using a nasal spray application.

To the best of our knowledge, this is the first case report on an adolescent with TRD using intranasally administered esketamin. Although the assessment of efficacy is complicated by comorbid diagnoses of autism and borderline personality, a case like M's is not atypical in child and adolescent psychiatry. Thus, given the dearth of research exploring ketamine use for TRD in the adolescent population, this case of an adolescent female receiving ready-made available nasal spray application of esketamine proves to be a first step in learning more about the potential benefits of this particular treatment method. While the intranasal application has been licensed for the use in TRD in adults, this drug is still off-label for use with adolescents. However, given the easy mode of administration, intranasal application of esketamine could lead to increased clinical use.

## Data availability statement

The original contributions presented in the study are included in the article/supplementary material, further inquiries can be directed to the corresponding author.

## Ethics statement

Ethical review and approval was not required for the study on human participants in accordance with the local legislation and institutional requirements. Written informed consent was obtained from the patient for the publication of this case report.

## Author contributions

HE and PP conceived the protocol. KD and HE executed the study. KS wrote the first draft of the paper. KN supervised the trial and contributed substantially to the paper. All authors read and approved the final version.
